# Toxic and essential elements in honeybee venom from Slovakia: Potential health risk to humans

**DOI:** 10.1016/j.heliyon.2024.e39282

**Published:** 2024-10-11

**Authors:** Rastislav Sabo, Martin Staroň, Lucia Sabová, Ivona Jančo, Marián Tomka, Július Árvay

**Affiliations:** a University of Veterinary Medicine and Pharmacy in Košice, Komenského 73, 041 81, Košice, Slovakia; b Research Institute for Animal Production Nitra, Institute of Apiculture Liptovský Hrádok, Dr. J. Gašperíka 599, 033 01, Liptovský Hrádok, Slovakia; c Slovak University of Agriculture, Research Centre Agrobiotech, Nitra, Tr. A. Hlinku 2, 949 76, Nitra, Slovakia; d Slovak University of Agriculture, Institute of Biotechnology, Nitra, Tr. A. Hlinku 2, 949 76, Nitra, Slovakia; e Slovak University of Agriculture, Institute of Food Science, Nitra, Tr. A. Hlinku 2, 949 76, Nitra, Slovakia

**Keywords:** Honeybee venom, Potentially toxic elements, Intoxication, Long-term risk assessment, Bioaccumulation

## Abstract

Honeybee venom is one of the natural substances produced by bees (*Apis mellifera*). Their venom gland produces venom which plays a defensive role. In this study a concentration of macro and trace elements (Ag, Al, As, Ba, Ca, Cd, Co, Cr, Cu, Fe, K, Li, Mg, Mn, Mo, Na, Ni, Sb, Se, Sr, Pb and Zn) in foragers′ and honeybees′ venom was analysed by axial inductively coupled plasma optical emission spectrometry (ICP OES) with good validation parameters to differentiate the element accumulation ability in honeybee venom. Cumulative ability for some elements (As, Al, Ba, Cr, Li, Mo, Pb, and Zn) in bee venom was clearly demonstrated. Oppositely, levels of macro elements (Ca, K, Mg and Na) in venom were several times lower compared to the levels detected in foragers. Moreover, PCA analysis of bee samples showed that Cr was associated with locality Košice, and Cd with locality Krompachy; both have rich industrial history. Since some of analysed elements are potentially toxic for humans, a risk assessment for bee-stung scenario was also calculated. A new way of exposure to potentially toxic elements via honeybee stung was showed in this study. Non-carcinogenic risk assessment for humans to selected toxic elements (As, Cd, Cr, Ni, and Pb) demonstrated acceptable risk and moreover the same we may conclude for potential carcinogenic risk for beekeepers exposed to As, Cd, Ni, and Pb via venom over their whole life.

## Highlights

• Some trace element levels in venom are several times higher compared to bees.
• Macro elements (Ca, Na, K, Mg) in venom are several times lower compared to adult bees.
• Positive association of Cr and Cd levels with localities with rich industrial history was showed.
• Non-carcinogenic risk assessment to toxic elements (Cd, Pb, As, Cr, Ni) demonstrated acceptable risk.
• Carcinogenic risk for humans to Pb, As, Cd, and Ni exposed via venom over 70 years is acceptable, too.

## 1 Introduction

Honeybees, under certain stressful conditions, are used to inject their venom into a victim′s body as a defensive mechanism developed over several thousand years of evolution. Honeybee venom (HBV) is produced by an acid gland (venom gland), which is an exocrine gland. The activity of the acid gland increases with the age of honeybees, with a peak in bees aged 14–21 days [1]. HBV is stored in the reservoir (sac) for later use when honeybees become foragers [2]. The bee venom gland produces raw bee venom, which contains a high proportion of water (80–85 %). Only approximately 148 μg of dry matter can be isolated from a single bee; the rest is composed of a wide range of proteins and peptide toxins [3,4]. However, a maximum content of 330 μg of dry matter from a single bee venom apparatus was isolated in a study done in the USA [5].

The composition of HBV depends on different factors related to the bee′s age, strain, caste, seasons, and methods of BV collection [6]. HBV is a complex mixture of biogenic peptides and amines with both immunological and pharmacological activities [[7], [8], [9], [10]]; more than 18 pharmacologically active compounds have been identified in bee venom [11]. After the stinging apparatus of the honeybee is torn from the body, 100 % of its contents are delivered within 60 s to the enemy′s body [12].

According to the World Allergy Organisation (WAO), insect venom is one of the most frequent inductors of anaphylactic reaction in humans [13]. Moreover, HBV allergy is the second most common form of allergy to Hymenoptera venom [10]. Children, beekeepers, and their other family members represent a group at risk who are more likely to be stung [14]. In Germany, the mean annual number of bee stings was 57.8 per active beekeeper [15], while in Mexico, the number of stings per month averaged 33 [16].

On the other hand, HBV has been used for a long time in traditional and modern medicine. Apitherapy, many times considered “an alternative therapy”, uses bee venom in the treatment of many human diseases [[17], [18], [19]], and moreover, there are many registered pharmaceutical formulations available on the global and European market as well [6]. In apitherapy, there are two basic ways to administer HBV into the patient′s body. Bee venom therapy uses sterile lyophilized venom, which is injected directly into the target tissue at various doses *in situ*, while bee sting therapy uses direct application via bee stinger [20,21]. For sensitive people, several sessions (about 12–20 times) are used to get the best results from their treatment [22].

There is limited information on the mineral element profile of HBV at that time. Kokot and Matysiak [23] demonstrated differences in the content in 32 HBV samples, commercial ones included, and Choinska et al. [24] developed a new laboratory voltammetric method using a hanging mercury drop (HMDE) electrode or PLA-C electrode for the determination of selected potentially toxic elements (Cd, Cu, Ni, Pb, and Zn) in HBV. Currently, it is common laboratory practice to use more sophisticated analytical procedures and instrumentations, that provide much greater sensitivity, accuracy and repeatability of results obtained from relevant bee matrices [25,26].

Specific physicochemical characteristic of potential toxic elements, including persistency and cumulation potential, can lead to harmful effects in living organisms and serious environmental risks. And hence, there is increased scientific interest to study the honeybee as a suitable bioindicator of environmental pollution in the last few years [[27], [28], [29]].

Trace concentrations of mineral elements present in HBVs may potentially control, trigger, and/or stop the biochemical pathways in living organisms [30]. Moreover, beekeepers practicing their hobby their whole lives as well as the medicinal use of HBV in apitherapy, may potentially put humans at risk from chronic exposure with potentially toxic element(s). Long-term exposure to cadmium, chromium, and arsenic may cause defects in DNA repair after the induction of oxidative stress in an organism and thus should be considered as potentially carcinogenic elements [31,32]. Exposure to lead may also disrupt the biological, neurological, and cognitive functions in the human bodies [33]. Exposure to high levels of nickel has been associated with hematotoxicity, immunotoxicity, genotoxicity, teratogenicity, and carcinogenicity [34].

The current study is aimed at determining the mineral element content of HBV and the resulting potential for chronic risk for humans. To observe and assess the potentially cumulative capacity of bee venom glands producing HBV, we used forager bees in mineral element analysis. Subsequently, measured element concentrations in bees and HBV were used in the bioaccumulation factors calculation.

## 2 Material and methods

### 2.1 Description of sampling areas

**Fig. 1:**
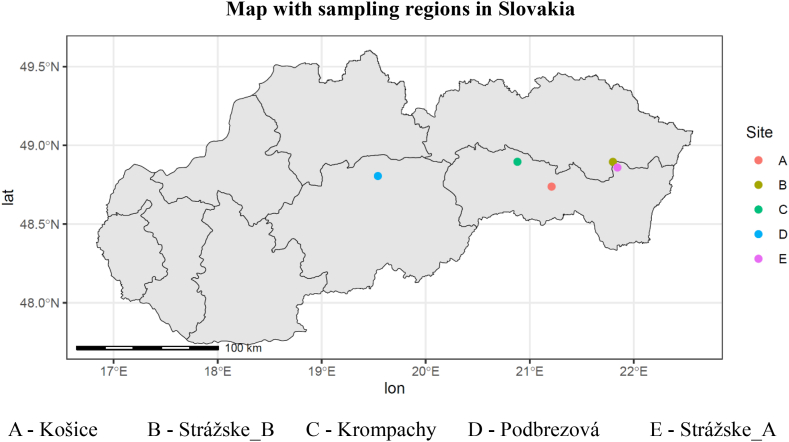
Map with sampling regions in Slovakia.

Sampling area A was an intraurban apiary localised in the north part of the city Košice, where an active steel factory and waste incineration plant have been present for a long time (12 km apart from the steel factory). Sampling areas B and E were intraurban apiaries, too. They are localised in the town of Strážske, with a distance of 0.2 km and 1.5 km from the chemical-producing plant, respectively. This plant has been active since 1952. Sampling area C was an extra urban apiary. The apiary is 2.5 km south of the steel factory in Krompachy. The last sampling area (D) was an extra urban apiary localized in the south-east part of village Podbrezová, where an active steel factory has been present for a long-time (1.4 km apart from the factory). The minimum distance of 2.0 km was only between intraurban localities in Strážske; other distances between sampling areas were ≥35 km (Fig. 1, Table S1).

### 2.2 Sampling

For the purpose of the current study, forager bees and HBV were collected between June 9, 2021, and June 16, 2021, from five heavily industrialised areas in Slovakia, aspiring to investigate “the worst case” scenario in our risk assessment. Sampling was always performed during sunny days between 10:00 a.m. and 02:00 p.m.

HBV was collected using a bee venom collection device (BeeWhisper, IGK Electronics Ltd., Bulgaria) using electric pulses for bee stimulation. During the collection, producer security recommendations to enhance the safety and protection of device users were fully followed. Forager bees at the age of ≥18 days are supposed to be more likely to be exposed to chemicals than younger bees. Foragers were collected at the hive entrance before venom collection. Each venom and bee sample represents a pool sample that originated from at least four beehives from the same apiary. The number of hives differed due to the different total number of hives at the respective apiary suitable for bee venom collection (it is linked with appropriate colony strength). The total amount of dry venom per colony is summarised in supplementary material (supplementary data; Table S1).

The amount of collected bee venom was sufficient for the next laboratory analysis. All the samples were stored in the freezer at −18 °C and sent within two days to the laboratory for further analysis. ICP OES analysis was performed at the Slovak University of Agriculture in Nitra, Slovakia.

### 2.3 Venom samples preparation

The frozen venom and bee samples were digested by high pressure microwave digestion. Approximately 0.1000 g (with a precision to 4 decimal places) of sample was weighed into PTFE digestion vessels. Consequently, 8 mL of HNO_3_ and 2 mL of H_2_O_2_ (trace purity, Sigma-Aldrich Chemie GmbH, Steiheim, Germany), was added directly to the PTFE vessels. The digestion procedure was carried out using pressure microwave digestion system ETHOS-One (Milestone, Srl., Italy) with the following parameters: I. ramp: 200 °C, 20 min; II. hold: 200 °C, 20 min, and cooling: 20 min. The mineralized sample solutions were filtered through a quantitative Munktell filter paper No. 390 (Munktell & Filtrak, Bärenstein, Germany) into 50 mL volumetric flasks and filled with double deionized water (ddH_2_O) to the final volume. Sample solutions were stored in polyethylene tubes until ICP OES analysis. Each sample was prepared in three replicates. Ultra-pure water ddH_2_O (18.2 MΩ cm^−1^, 25 °C) was treated in a Simplicity 185 purification (Millipore SAS, Molsheim, France) and was used in all cases.

### 2.4 Determination of elements

Elemental analysis was carried out on an Agilent ICP OES spectrometer 720 (Agilent Technologies Inc., Santa Clara, CA, USA) with axial plasma configuration and with an auto-sampler SPS-3 (Agilent Technologies, GmbH, Germany). Detailed experimental conditions were set as follows: RF power: 1.75 kW; plasma gas flow: 16.0 L min^−1^; auxiliary gas flow: 1.50 L min^−1^ and nebulizer gas flow: 0.85 L min^−1^ and CCD detector temperature −35 °C. Signal accusation time 3 × 3 s for three replicates. Calibration of the analytical method ICP OES was realized using mixed standard TraceCert ICP 5 (Sigma Aldrich, GmbH, Steiheim, Germany), which was diluted to the three calibration levels (I.: 0.0475 mg kg^−1^; II.: 0.0950 mg kg^−1^; III.: 0.190 mg kg^−1^). Argon and carbon were used as internal standard elements. ERMVR-CE278k (mussel tissue; IRMM, Belgium) and blank were used for quality measurements control. The recovery values, limits of detection, limits of quantification, determination wavelengths, and linearity of calibration curves are presented in Table S2 (Supplementary data). For non-certified elements, the recovery was defined in the standard addition method.

### 2.5 **Human health risk assessment** (carcinogenic and non-carcinogenic risks)

In the current study, the carcinogenic and non-carcinogenic risks posed by the most toxic elements detected in bee venom were assessed by calculating the target hazard quotient (HQ), while the hazard index (HI) was used to approximate the chronic-toxic risk.

Because there is not an exact risk assessment scheme available for bee-stung scenario at this time, this approach must be taken as an illustrative risk assessment, and additional research is needed to support such an approach. In our assumption, an average of 396 bee stings per year was used [16], with the maximum documented bee venom content of 330 μg of dry matter in a sac [5]. The European population somatometric value of 70 kg body weight for adult humans was used in our calculation, children are not expected to be exposed chronically over the year(s) in this study.

In our risk assessment calculation, we followed USEPA recommendations [35,36] and recently published works [37,38]. Based on the aforementioned factors, the following equation was used:

(1) $$EDI=\frac{VIR\times C}{BW}$$

Adequately, the food intake ratio (FIR) in this case was replaced by a venom intake ratio (VIR); the calculation approach is described in the text. The non-carcinogenic risk (HQs and HI) was calculated using the following equations:

(2) $$HQ=\frac{EDI\;x\;EF\;x\;ED}{HBGV\;x\;AT}$$

(3) $$HI=\sum_{k=1}^{n}HQ_{k}{=HQ}_{1}+HQ2+HQ3+\ldots +HQn$$

Where:

EDI: estimated daily intake (μg kg^−1^ day^−1^),

VIR: venom intake rate = 358.03 μg venom/adult human/day; averaged 33 stings per month [16] with max. of 330 μg of dry matter [5],

C: concentration of the analysed element (μg kg^−1^),

EF: exposure frequency = 365 days per year,

ED: exposure duration = 27 years,

BW: average body weight 70 kg for adults, and.

AT: average exposure time = 365 days ∗ ED.

The health-based guidance values (HBGVs) of the most toxic elements were obtained from the European Safety Authority [[39], [40], [41]] and US-EPA [42], and they were equal to 0.3, 0.36, 3.0, 20.0, and 2.0 μg kg^−1^ b.w. day^−1^ for As, Cd, Cr, Ni, and Pb, respectively.

In our carcinogenic risk (CR) calculation posed by the most toxic metal elements detected in HBV, we used two factors, EDI and CPFo (cancer potency factor); EDI values were based on equation (1), with ED being equal to 70 years instead of 27. CPFo values were retrieved from the Office of Environmental Health Hazards Assessment [43], the Risk Assessment Information System [44], and the United States Environmental Protection Agency [42].

According to IARC [45], As, Cd, Cr, Ni, and Pb are classified as carcinogenic or probable carcinogenic to humans, with As, Cd, Cr, and Ni belonging to category 1 of undesired trace elements for cancer. Considering the stated facts, the carcinogenic risk (CR) of these elements was calculated considering their respective CPFo values. At this point, it is worth mentioning, that the CPFo value for Cd has not been set, and thus CR could not be calculated for this element. Carcinogenic risk was computed by using the following two equations:

(4) $$CR_{k}=EDI\times CPF_{o\;}\times EF\times ED/AT$$

(5) $$CR=\sum_{k=1}^{n}CR_{k}$$

### 2.6 **Bioaccumlation factors** (BAFs)

The bioaccumulation factor is one of the most common toxicological indicators, which represents the influence of mineral element content distribution in the bee′s body on the venom content and objectively reflects the ability of the venom gland to potentially “uptake” mineral elements from the body. The calculation of BAFs is expressed as:

(6) $$\text{BAFs}=C_{\text{venom}}/C_{\text{bees}}$$

where C_venom_ and C_bees_ are the mineral element concentrations analysed in venom and body, respectively. The values measured below the LOQ were excluded from the BAF calculation.

### 2.7 Statistical analysis

The data in this study were processed and analysed using the statistical program R [46]. To compare the potentially toxic elements content in forager bees and venom, a generalised linear model (GLM) was fitted for the data with a Gamma distribution using log line.

To reduce the number of variables in our data set, a principal component analysis (PCA) analysis was performed using the MASS package [47] and factoextra [48]. From the measured concentrations (initial variables) for As, Cd, Cr, Ni, and Pb, after their standardisation, a separate covariance matrix for honeybees and for bee venom was computed. In the next step, the eigenvectors and eigenvalues of the covariance matrix to identify the principal components were computed. Finally, the data were visualised using the ggplot2 package [49].

## 3 Results and discussion

HBV was collected at the hive entrance using a bee venom collection device (BeeWhisper). Each venom sample was a pool sample originated from at least four beehives from the same apiary. The total amount of obtained venom in dry form strongly varied among hives (14.9–77.5 mg dry venom), maybe due to different colony strengths (Table S1, Supplementary data).

### 3.1 Concentrations of elements and bioaccumlation factors

In the current study, macro (Ca, K, Mg, and Na) and trace elements (As, Ag, Al, Ba, Cd, Co, Cr, Cu, Fe, Li, Mn, Mo, Ni, Pb, Sb, Se, Sr, and Zn), including potentially toxic elements (As, Cd, and Pb), were analysed with optimised analytical method in two different matrices from heavily industrialised areas in Slovakia. Mean mineral element concentrations with respective standard deviation values (±SD) and maximum measured concentrations in venom and forager bees are listed in Table 1.

**Table 1:** Mineral element profile in bees and venom (mg kg^−1^) and resulted bioaccumlation factors.

Element	Sample	Bees (mean ± SD)						Venom (mean ± SD)						bioaccumlation factors; venom/bees				
	locality	A	B	C	D	E	maximum measured concentrations	A	B	C	D	E	maximum measured concentrations	A	B	C	D	E
**Ag**		0.026 ± 0.038	0.010 ± 0.019	0.010 ± 0.018	0.007 ± 0.012	0.009 ± 0.014	0.150	0.020 ± 0.024	0.539 ± 0.020	0.000 ± 0.000	0.006 ± 0.008	0.018 ± 0.021	0.557	0.764	55.529	- - -	0.857	2.073
**Al**		13.606 ± 7.621	10.287 ± 1.649	50.825 ± 68.508	10.877 ± 10.493	4.759 ± 2.759	198.783	84.336 ± 0.473	91.166 ± 0.368	75.849 ± 0.825	96.986 ± 0.865	44.176 ± 0.173	97.829	6.199	8.862	1.492	8.916	9.282
**As**		0.453 ± 0.798	0.420 ± 0.670	0.377 ± 0.517	0.406 ± 0.575	0.547 ± 0.781	3.304	0.326 ± 0.565	0.864 ± 1.497	1.039 ± 1.800	0.000 ± 0.000	0.487 ± 0.843	**3.118**	0.719	2.059	2.759	- - -	0.890
**Ba**		1.329 ± 0.522	1.325 ± 0.714	2.713 ± 1.451	0.598 ± 0.349	0.492 ± 0.433	5.565	2.979 ± 0.004	1366.293 ± 2.823	5.588 ± 0.021	4.738 ± 0.007	10.235 ± 0.027	1368.790	2.241	1031.061	2.059	7.926	20.805
**Ca**		796.284 ± 86.049	1140.069 ± 136.759	696.166 ± 174.510	440.086 ± 118.068	802.348 ± 189.151	1271.264	472.33 ± 9 2.524	777.832 ± 1.667	386.698 ± 2.139	369.796 ± 3.170	366.061 ± 0.300	779.522	0.593	0.682	0.555	0.840	0.456
**Cd**		0.000 ± 0.000	0.000 ± 0.000	0.015 ± 0.044	0.000 ± 0.000	0.000 ± 0.000	0.182	0.000 ± 0.000	1.923 ± 0.158	0.000 ± 0.000	0.000 ± 0.000	0.000 ± 0.000	**2.059**	- - -	- - -	- - -	- - -	- - -
**Co**		0.037 ± 0.063	0.027 ± 0.044	0.009 ± 0.020	0.020 ± 0.023	0.027 ± 0.073	0.301	0.009 ± 0.016	0.057 ± 0.052	0.000 ± 0.000	0.006 ± 0.010	0.011 ± 0.019	0.103	0.242	2.123	- - -	0.299	0.411
**Cr**		0.519 ± 0.479	0.043 ± 0.054	0.020 ± 0.036	0.335 ± 0.223	0.010 ± 0.025	1.615	1.311 ± 0.082	3.834 ± 0.064	3.575 ± 0.074	3.810 ± 0.017	0.510 ± 0.067	**3.907**	2.523	88.807	177.748	11.367	49.609
**Cu**		16.864 ± 2.905	13.322 ± 0.479	15.390 ± 2.721	7.867 ± 0.953	10.429 ± 4.241	19.486	3.004 ± 0.012	3.883 ± 0.020	4.563 ± 0.017	1.586 ± 0.021	0.881 ± 0.021	4.575	0.178	0.291	0.296	0.202	0.085
**Fe**		177.215 ± 39.706	85.893 ± 3.125	98.740 ± 13.293	58.029 ± 23.167	58.990 ± 20.059	238.562	146.453 ± 0.799	537.149 ± 1.990	219.976 ± 0.670	170.320 ± 0.454	58.589 ± 0.037	539.416	0.826	6.254	2.228	2.935	0.993
**K**		4505.032 ± 702.430	3866.213 ± 378.361	4738.932 ± 835.855	3218.339 ± 502.720	3217.431 ± 582.904	5540.360	2611.973 ± 5.932	1584.217 ± 4.821	3187.411 ± 12.948	2573.583 ± 16.449	1221.451 ± 5.878	3199.353	0.580	0.410	0.673	0.800	0.380
**Li**		0.043 ± 0.041	0.013 ± 0.004	0.018 ± 0.014	0.011 ± 0.006	0.009 ± 0.010	0.199	0.072 ± 0.006	0.052 ± 0.003	0.056 ± 0.008	0.062 ± 0.001	0.040 ± 0.009	0.077	1.663	4.041	3.027	5.536	4.422
**Mg**		685.794 ± 119.967	948.423 ± 243.472	702.172 ± 173.908	481.501 ± 88.482	611.758 ± 123.014	1170.943	291.410 ± 1.101	329.871 ± 0.895	318.941 ± 1.112	304.05 ± 0.550	179.866 ± 0.713	330.900	0.425	0.348	0.454	0.631	0.294
**Mn**		66.436 ± 24.171	38.885 ± 18.859	60.856 ± 18.450	21.660 ± 2.620	22.948 ± 12.802	110.781	4.737 ± 0.021	26.257 ± 0.078	8.558 ± 0.001	7.445 ± 0.009	2.276 ± 0.009	26.340	0.071	0.675	0.141	0.344	0.099
**Mo**		0.054 ± 0.089	0.000 ± 0.000	0.000 ± 0.000	0.020 ± 0.033	0.000 ± 0.001	0.282	1.443 ± 0.183	0.248 ± 0.219	1.215 ± 0.347	0.501 ± 0.002	0.000 ± 0.000	1.652	26.726	- - -	- - -	25.154	- - -
**Na**		277.784 ± 58.917	321.435 ± 23.515	275.598 ± 55.909	159.023 ± 31.356	210.605 ± 56.603	343.551	245.099 ± 0.616	174.827 ± 0.669	209.178 ± 1.285	158.700 ± 0.771	167.651 ± 0.976	245.752	0.882	0.544	0.759	0.998	0.796
**Ni**		1.824 ± 1.184	1.056 ± 0.569	3.264 ± 1.430	0.822 ± 0.283	0.608 ± 0.465	6.665	8.434 ± 0.474	1.267 ± 0.394	1.107 ± 0.735	1.037 ± 0.306	0.590 ± 0.047	**8.963**	4.623	1.200	0.339	1.262	0.970
**Pb**		0.009 ± 0.038	0.000 ± 0.000	0.218 ± 0.404	0.233 ± 0.441	0.000 ± 0.000	1.304	68.672 ± 0.449	52.630 ± 0.238	37.493 ± 0.943	56.450 ± 1.081	16.144 ± 0.548	**69.140**	7592.709	- - -	171.817	241.857	- - -
**Sb**		0.291 ± 0.750	0.474 ± 0.558	0.390 ± 0.498	0.220 ± 0.298	0.354 ± 0.491	3.133	0.285 ± 0.494	1.091 ± 0.694	1.031 ± 0.937	0.135 ± 0.233	1.355 ± 1.252	2.468	0.979	2.300	2.643	0.612	3.826
**Se**		0.240 ± 0.700	0.280 ± 0.446	0.276 ± 0.527	0.040 ± 0.090	0.555 ± 0.969	3.745	1.100 ± 1.210	0.123 ± 0.212	0.000 ± 0.000	0.234 ± 0.406	0.000 ± 0.000	2.397	4.585	0.437	- - -	5.874	- - -
**Sr**		1.573 ± 0.274	2.402 ± 0.106	0.434 ± 0.294	0.699 ± 0.190	1.309 ± 0.780	2.503	1.187 ± 0.003	20.392 ± 0.043	0.146 ± 0.003	0.229 ± 0.003	0.095 ± 0.004	20.440	0.755	8.489	0.336	0.328	0.073
**Zn**		105.746 ± 31.308	50.732 ± 2.618	58.078 ± 9.693	39.684 ± 18.324	41.789 ± 12.436	150.908	1562.420 ± 16.405	3305.000 ± 23.300	1672.719 ± 2.533	1453.391 ± 4.294	668.198 ± 2.403	3322.553	14.775	65.146	28.801	36.624	15.990

- - - not determined.

values **in bold** are used in risk assessment.

Samling localities: A – Košice; B - Strážske_B; C – Krompachy; D – Podbrezová; E − Strážske_A.

The majority of detected levels of macro- and trace elements in forager bees are in the range of recently published results [28]. Their maximum measured levels of 161, 458, 54, 830, and 1735 μg kg^−1^ in forager bees originated from eastern Slovakia, compared to 3304, 182, 1615, 6665, and 1304 μg kg^−1^ obtained in this study for As, Cd, Cr, Ni, and Pb, respectively, were almost in the same range except for elements As, Cr, and Ni (Table 1). The observed discrepancy in As, Cr, and Ni concentrations between these two studies is unknown.

**Fig. 2:**
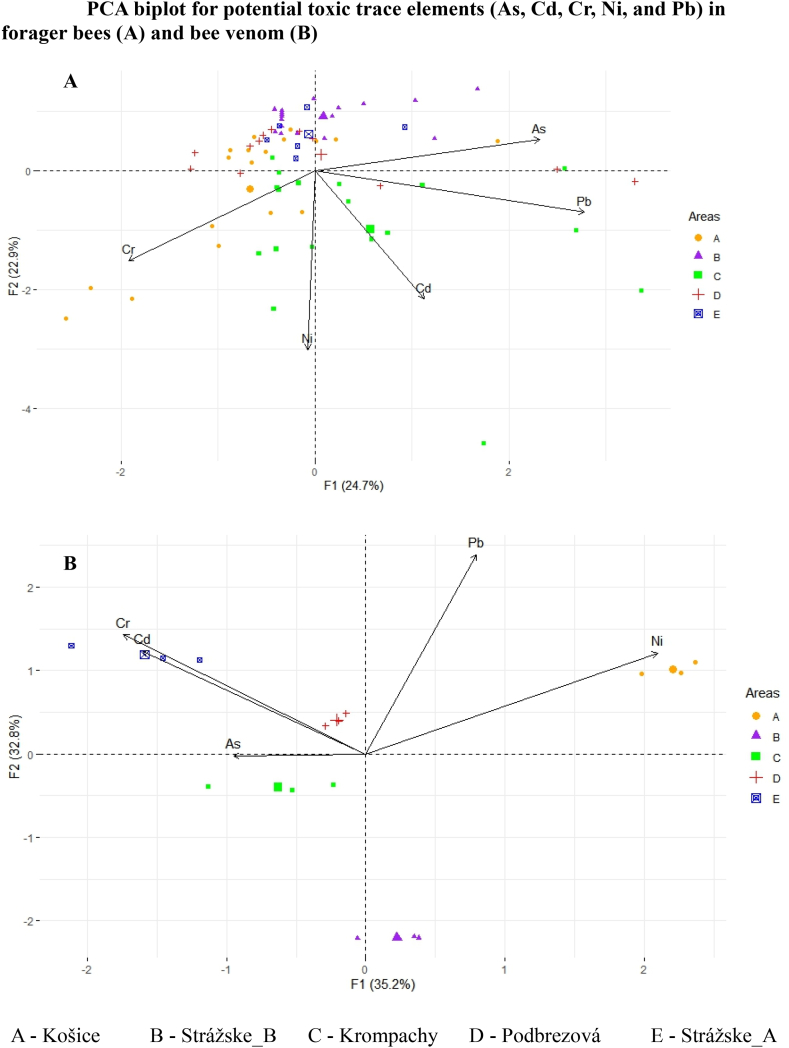
PCA biplot for potential toxic trace elements (As, Cd, Cr, Ni, and Pb) in forager bees (A) and bee venom (B).

According to some published studies, limits of 52, 40, 100, and 300 μg kg^−1^ for Cd, Cr, Ni, and Pb, respectively, in honeybees indicate low metal contamination of terrestrial areas [50,51]. Surprisingly, based on the mean measured results in forager bees for Ni in this study, all sampling areas indicated high pollution with nickel (calculated on a wet weight basis). Levels of chromium measured in forager bees originated from localities A (Košice), D (Podbrezová), and E (Strážske_A) surpassed the above-mentioned limit of 52 μg kg^−1^. A lower terrestrial area pollution picture was shown when Pb and Cd levels were compared. Pb levels exceeded the limit in localities C (Krompachy) and D (Podbrezová), whereas Cd levels only exceeded the limit in locality 5. (Krompachy). To confirm this finding and to find some relationship between mean measured levels of the elements and sampling areas, we decided to apply a PCA analysis for toxic trace elements (As, Cd, Cr, Ni, and Pb) in forager bees as well as in HBV for completeness (Fig. 2).

When PCA analysis (Fig. 2A) was applied to potentially toxic elements in forager bees (for a scree plot of percentage of explained variance, see Figs. S1 and S2, and for the Importance of components, Rotation (Eigenvectors) and PC scores Tables S3 and S4; Supplementary data), Cr was associated with sampling area A (Košice), and Cd with sampling area C (Krompachy). Both areas have rich industrial history with steel and copper factories, respectively. In the case of venom (Fig. 2B), we got a slightly different picture; Ni was associated with sampling area A (Košice), Cr and Cd were associated with sampling area E (Strážske_A), and As was associated with sampling area C (Krompachy).

At that time, only one scientific study dealing with the full element content profile of HBV is available. The content of macro- and trace elements in HBV was studied using ICP MS by Kokot and Matysiak [23] in Poland. Generally, we can conclude that most of the obtained element levels in our study are comparable to the element concentrations observed in Kokot and Matysiak [23], except for the levels of some potentially toxic elements (As, Cd, and Pb). Their measured levels of 0.3, 0.11, and 5.6 mg kg^−1^, compared to 3.1, 2.1, and 69.1 mg kg^−1^ obtained in this study for As, Cd, and Pb, respectively, were at least ten times lower. Vice versa, levels of nickel and chromium were twice lower in our study compared to the results from Kokot and Matysiak [23]. Using the voltammetric method, Choinska et al. [24] obtained maximum levels of 4.54 ± 0.63, 26.2 ± 2.0, and 6.29 ± 0.63 mg kg^−1^ for Cd, Ni, and Pb in HBV samples, respectively. Comparing these concentrations to our results, the maximum measured level of lead (69.1 mg kg^−1^) in this study is almost ten times higher, while levels of the remaining two elements (1.9 mg kg^−1^ for Cd and 8.4 mg kg^−1^ for Ni) in our study were almost twice lower. These differences in metal concentration levels may be probably explained by the seasonal and year-to-year differences in element content profiles in HBV samples observed in Kokot and Matysiak [23] and the different samples’ origins linked to unknown pollution history. Finally, we may conclude, that the observed higher levels of potentially toxic elements in HBV in this study confirmed our right choice to collect samples in heavily industrialised areas in Slovakia, thus aspiring to evaluate “the worst case” scenario in our chronic risk assessment.

**Fig. 3:**
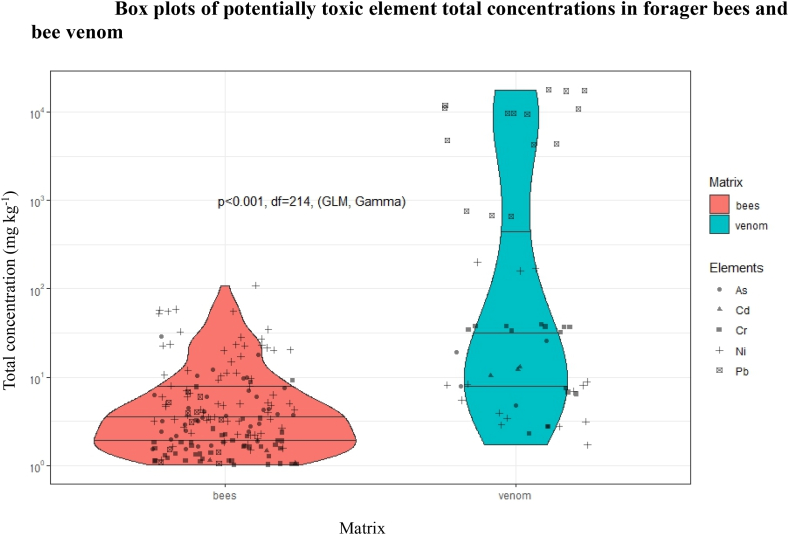
Box plots of potentially toxic element total concentrations in forager bees and bee venom.

Further, by comparing the total concentration of potentially toxic elements (As, Cd, Cr, Ni, and Pb) between both analysed matrices, it seems that honeybee venom contains more than double the total concentration of potentially toxic elements, statistically significant at p < 0.001 (Fig. 3).

**Fig. 4:**
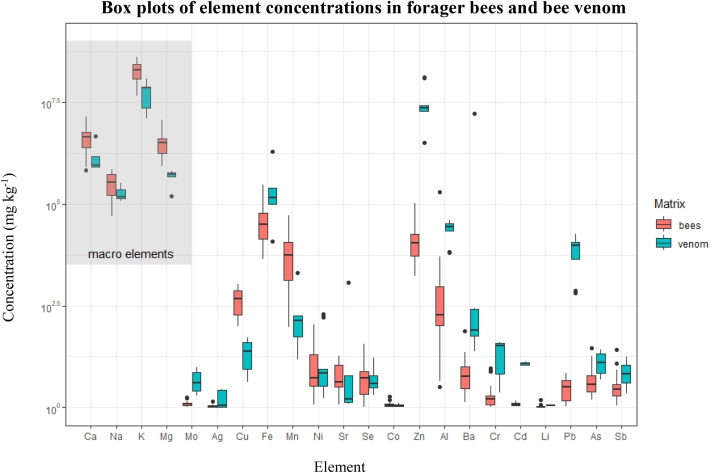
Box plots of element concentrations in forager bees and bee venom.

When the bioaccumulation factors for Ca, K, Mg, and Na in venom and adult bees were applied in this study, honeybee venom seemed to contain lower concentrations of macro elements (Table 1 and Fig. 4).

Since all measured Cd and some Pb concentrations were below the LOQ in forager bees and/or HBV samples, the majority of bioaccumulation factors were not determined for these elements (Table 1). But looking at other trace element levels in HBV and foragers in Table 1 and Fig. 4, some elements tended to cumulate in the venom gland.

Almost all bioaccumulation factors for molybdenum, chromium, zinc, aluminium, barium, lithium, lead, and arsenic are higher than one, indicating their higher cumulative ability in bee venom (Fig. 4). Among them, potentially toxic elements (As, Cr, and Pb) showed the highest observed bioaccumulation factors (Table 1), giving us an assumption of potentially non-carcinogenic and carcinogenic risks for beekeepers who are constantly exposed to these toxic elements while practicing beekeeping over the years.

### 3.2 Human exposure risk

Risk assessment should be based on the worst-case scenario, and for that reason, the maximum detected concentrations of potentially toxic elements were used in our assumption. Moreover, for the calculation of the EDI at the worst-case scenario, the maximum average of 33 stings per month [16] with the maximum of 330 μg of dry matter [5] were used in our risk assessment.

#### 3.2.1 Non-carcinogenic risk for humans

According to US-EPA [42], the risk assessment for humans is acceptable if the HQ value for a specific element and the HI value for a mixture of elements are below the trigger value of 1, while HQ values and HI ≥ 1 indicate potentially increased health risk. HQ values for the most toxic elements (As, Cd, Cr, Ni, and Pb) were calculated in HB venom for adults in this study. They are presented in Table 2.

**Table 2:** Non-carcinogenic risk assessment for adult humans.

matrix	element	maximum measured concentration (μg kg^−1^)	kg venom day^−1^	EDI (μg kg^−1^day^−1^)	HBGVs (μg^−1^kg^−1^day^−1^)	HQ/adults
**venom**	**As**	3118	3.58027E-07	0.0000148835	0.30	4.961172E-05
	**Cd**	2059	3.58027E-07	0.0000098290	0.36	2.730290E-05
	**Cr**	3907	3.58027E-07	0.0000186508	3.00	6.216947E-06
	**Ni**	8963	3.58027E-07	0.0000427867	20.00	2.139333E-06
	**Pb**	69140	3.58027E-07	0.0003300535	2.00	1.650268E-04
**HI**						2.502977E-04

∗ maximum average of 33 stings per month according to Becerril-Angeles and Nuñez-Velázquez [16] with max. of 330 μg of dry matter according to Schumacher et al. [16].

Obtained results showed that all of the calculated HQ and HI values for selected elements for adults were well below the trigger value of 1, indicating an acceptable non-carcinogenic risk for beekeepers long-term exposed via HBV. Vice versa to this finding, HQ values calculated for As, Cd, and Cr (adults and children) were above the threshold value of 1, indicating that Greek pollen samples can pose a non-carcinogenic risk after long-term consumption in a diet [38].

#### 3.2.2 Carcinogenic risk for humans

The carcinogenic risk for the most toxic elements (As, Cr, Ni, and Pb) for adult humans was calculated, too. The CR value for the carcinogenic risk refers to the probability of an individual to develop any type of cancer from lifetime exposure. Beekeeping is considered a highly skilled form of animal husbandry, and many times people practice this hobby their whole lives. Thus, the exposure of practicing beekeepers to potentially toxic elements via HBV may be considered justifiable.

Since mercury was not analysed in this study, and moreover, the CPFo value for Cd has not been assigned (see Material and Methods), the CRs could not be calculated for these two elements in our carcinogenic risk assessment. The results for adults are presented in Table 3.

**Table 3:** Carcinogenic risk assessment for adult humans.

matrix	element	concentration (μg kg^−1^)	EDI (μg kg^−1^ day^−1^)	CPFo (mg kg^−1^ day^−1^)^a^	CRk/adults
**venom**	**As**	3118	0.0000148835	1.5000	2.23253E-08
	**Cr**	3907	0.0000186508	0.5000	9.32542E-09
	**Ni**	8963	0.0000427867	0.9100	3.89359E-08
	**Pb**	69140	0.0003300535	0.0085	2.80545E-09
**CR**	**Ʃ**				7.3392E-08

a CPFo values obtained from the Office of Environmental Health Hazards Assessment [43], the Risk Assessment Information System [44], and the United States Environmental Protection Agency [42].

According to USEPA CR values, <1 × 10^−6^ can be regarded as negligible, and values in the range from 1 × 10^−6^ to 1 × 10^−4^ represent an acceptable risk [42]. Overall, the CR values obtained for all toxic elements (As, Cr, Ni, and Pb) in this study demonstrated negligible risk for adults (Table 3). Moreover, the estimated daily venom intake (EDI) of 1.49 × 10^−5^ μg As kg^−1^ bw per day in this study (Table 3) is much lower than the reference point of 0.06 μg iAs kg^−1^ bw per day proposed by the EFSA′s CONTAM Panel, which should also be considered applicable for chronic kidney disease, respiratory disease, lung cancer, bladder cancer, skin lesions, stillbirth, spontaneous abortion, infant mortality, and neurodevelopmental effects [52]. Vice versa to our findings, the majority of calculated CRk values for As, Cr, and Ni exceeded the threshold value of 1 × 10^−4^ assessing the carcinogenic risk of Greek′ bee pollen samples for adults and children [38], but in this case, differences in way of exposure (dietary way of exposure) must be taken into consideration.

In this study, we showed a new way of exposure to potentially toxic elements via honeybee stung. Irrespectively, the likely unrealistic worst-case bee-stung scenario for beekeepers exposed over the years, should be interpreted with caution. Possible interactions among elements together with pesticide residue interactions give us an assumption of potentially harmful effects on human health [[53], [54], [55]]. Moreover, depending on the level and duration of exposure, certain mineral elements can produce harmful toxic effects in living organisms even at very low concentrations, which have been associated with various symptoms, including toxic effects in nervous, lungs, kidney, liver, and immune system, diabetes, epigenetic changes in DNA expression, renal dysfunction, hypertension, or even death [[56], [57], [58], [59]]. At the cellular level, potentially toxic elements (As, Cd, Cr, Hg, and Pb) disrupt differentiation, growth, proliferation, damage-repairing processes, and apoptosis [32].

CR values of 2.23253E-08 and 3.89359E-08 for As and Ni, respectively, in this study are the highest calculated values in our carcinogenic risk assessment for adult humans (Table 3), but on the other hand they are well below the set trigger threshold of 1 × 10^−6^ [42]. Both elements are classified as carcinogenic or probable carcinogenic to humans, belonging to category 1 of undesired trace elements for cancer [45]. The main route of exposure of As is absorption from the small intestine; other known routes are inhalation and skin contact [60]. Toxicity of arsenic is mainly related to the dysfunctions of numerous enzymatic cycles in exposed organisms, from which an inhibition of sulfhydryl group containing enzymes and the pyruvate dehydrogenase were well described [61]. Element Ni plays an important role in the red blood cell synthesis and may be considered a crucial element for activation of some enzyme systems [62]. Its main exposure routes are via inhalation, ingestion, and dermal absorption, while the nervous system is one of the main target organs for Ni toxicity after long-term exposure to higher concentrations [63]. On the other hand, the presence of macroelements in diet is essential for the physiological function and growth of the human body [64].

However, the reported concentrations and resulted BCFs in this study should be interpreted with caution. Using more sophisticated analytical procedures and instrumentations [25,26] in this study could provide us much better analysis sensitivity and thus BCF values for more elements could be calculated (Table 1). But finally, the results of this study showed the necessity for concomitant chemical diversity monitoring to decipher possible interactions among them, as some studies have pointed out [[65], [66], [67]].

## 4 Conclusion

In the current study, we focused on determining the element content profile in bees and venom using the ICP OES method. The analytical method used provided us with high-quality results that complement the relatively little published issue, especially when it comes to bee venom.

The obtained element profile is mostly in line with already published data for adult bees and bee venom. Our results showed that some trace element levels in HBV are several times higher compared to forager bees, thus indicating its cumulative ability in the venom gland, e.g., the maximum measured concentration of lead in HBV was 69.1 mg kg^−1^ compared to 1.3 mg kg^−1^ in bees. On the other hand, the levels of macroelements (Ca, K, Mg, and Na) detected in HBV were several times lower compared to adult bees.

Applied PCA analysis showed that measured levels of Cr in bees were associated with sampling area A (Košice), and Cd levels with sampling area C (Krompachy). Venom data analysis showed that Ni was associated with sampling area A (Košice), Cr and Cd were associated with sampling area E (Strážske_A), and As was associated with sampling area C (Krompachy), thus confirming their rich industrial history.

Some beekeepers practice beekeeping as a hobby their whole lives, and thus their exposure to potentially toxic elements via bee-stung scenario can be considered justifiable. Despite non-carcinogenic and carcinogenic risk assessment for selected potentially toxic elements (As, Cd, Cr, Ni, and Pb) demonstrated acceptable risk for humans, a new way of long-term exposure to potentially toxic elements via honeybee stung described in this study should be taken as a relevant in the future.

Moreover, the potential risks of low levels of metal elements present in HBV in combination with various chemicals (residues of pesticides included) and their possible interactions, which may interfere with the biochemical pathways in the body, should not be underestimated, and thus further research is deemed necessary in this field.

## CRediT authorship contribution statement

**Rastislav Sabo:** Supervision, Resources, Methodology. **Martin Staroň:** Methodology, Funding acquisition. **Lucia Sabová:** Writing – review & editing, Writing – original draft. **Ivona Jančo:** Validation, Investigation, Data curation. **Marián Tomka:** Validation, Data curation. **Július Árvay:** Supervision.

## Data availability statement

Data will be made available on request.

## Ethical approval

Not applicable.

## Consent to participate

All authors agreed with the content, all authors gave explicit consent to submit, and we obtained consent from the responsible authorities at the institute/organization where the work was carried out, before the work was submitted.

## Consent for publication

All authors gave the publisher the permission to publish the work.

## Funding information

This research was supported by the Slovak Grant Agency APVV-21-0185, VEGA 1/0161/23 and by the National Reference Laboratory for Pesticides of University of Veterinary Medicine and Pharmacy in Košice, Slovakia (sampling part of this study).

## Declaration of competing interest

The authors declare that they have no known competing financial interests or personal relationships that could have appeared to influence the work reported in this paper.
