# Supplementary material for: Toxic and essential elements in honeybee venom from Slovakia: Potential health risk to humans
Source: Heliyon. 2024 Oct 11;10(20):e39282. doi: 10.1016/j.heliyon.2024.e39282 (PMC11530785; doi:10.1016/j.heliyon.2024.e39282)
Supplement: Multimedia component 1 [file mmc1.docx]

**Table S1.** **Other sampling details; the total amount of dry venom per colony**

| Sampling area | **A** (Košice) | **B** (Strážske_B) | **C** (Krompachy) | **D** (Podbrezová) | **E** (Strážske_A) |
| --- | --- | --- | --- | --- | --- |
| GPS | 48°44'18.85"S  21°14'54.44"V | 48°52'7.47"S  21°50'40.14"V | 48°54'50.46"S  20°52'25.95"V | 48°48'32.75"S  19°31'32.91"V | 48°52'19.51"S  21°49'32.62"V |
| Date of sampling | 13.6.2021 | 9.6.2021 | 9.6.2021 | 16.6.2021 | 11.6.2021 |
| **Number of hives** | **6** | **4** | **7** | **4** | **5** |
|  | 69.7 | 61.2 | 77.5 | 60.5 | 47.0 |
|  | 39.3 | 67.3 | 29.0 | 68.9 | 14.9 |
|  | 44.9 | 53.1 | 24.0 | 42.1 | 46.5 |
|  | 42.4 | 67.5 | 15.2 | 53.9 | 16.0 |
|  | 52.0 |  | 49.3 |  | 20.3 |
|  | 22.4 |  | 15.1 |  |  |
|  |  |  | 33.1 |  |  |
