# Supplementary material for: Toxic and essential elements in honeybee venom from Slovakia: Potential health risk to humans
Source: Heliyon. 2024 Oct 11;10(20):e39282. doi: 10.1016/j.heliyon.2024.e39282 (PMC11530785; doi:10.1016/j.heliyon.2024.e39282)
Supplement: Multimedia component 2 [file mmc2.docx]

**Table 2S. Details of quality control of ICP OES analysis**

| **Elements** | **Wavelengths**  **(nm)** | **LOD**  **(mg kg^-1^)** | **LOQ**  **(mg kg^-1^)** | **Recovery (%)** | **Linearity** |
| --- | --- | --- | --- | --- | --- |
| **Ag** | 338.289 | 0.0036 | 0.0119 | 100 | 0.9996 |
| **Al** | 396.152 | 0.0071 | 0.0234 | 98 | 0.9995 |
| **As** | 188.980 | 0.0135 | 0.0446 | 93 | 0.9997 |
| **Ba** | 455.403 | 0.0033 | 0.0109 | 98 | 0.9995 |
| **Ca** | 422.673 | 0.0682 | 0.2251 | 97 | 0.9998 |
| **Cd** | 214.468 | 0.0004 | 0.0013 | 93 | 0.9997 |
| **Co** | 238.892 | 0.0018 | 0.0059 | 96 | 0.9999 |
| **Cr** | 284.325 | 0.0112 | 0.0370 | 95 | 0.9996 |
| **Cu** | 324.754 | 0.0030 | 0.0100 | 102 | 0.9997 |
| **Fe** | 238.204 | 0.0011 | 0.0036 | 98 | 0.9998 |
| **K** | 766.490 | 0.5052 | 1.4472 | 99 | 0.9994 |
| **Li** | 670.783 | 0.0012 | 0.0040 | 94 | 0.9994 |
| **Mg** | 280.270 | 0.0003 | 0.0010 | 101 | 0.9999 |
| **Mn** | 257.610 | 0.0003 | 0.0010 | 95 | 0.9999 |
| **Mo** | 204.598 | 0.0041 | 0.0135 | 99 | 0.9999 |
| **Na** | 818.326 | 0.1804 | 0.5953 | 96 | 0.9999 |
| **Ni** | 221.647 | 0.0017 | 0.0056 | 94 | 0.9995 |
| **Pb** | 220.353 | 0.0317 | 0.1046 | 97 | 0.9996 |
| **Sb** | 206.834 | 0.0144 | 0.0475 | 92 | 0.9998 |
| **Se** | 196.026 | 0.0056 | 0.0185 | 99 | 0.9998 |
| **Sr** | 421.552 | 0.0021 | 0.0069 | 94 | 0.9999 |
| **Zn** | 213.856 | 0.0069 | 0.0228 | 102 | 0.9999 |
