# Supplementary material for: Toxic and essential elements in honeybee venom from Slovakia: Potential health risk to humans
Source: Heliyon. 2024 Oct 11;10(20):e39282. doi: 10.1016/j.heliyon.2024.e39282 (PMC11530785; doi:10.1016/j.heliyon.2024.e39282)
Supplement: Multimedia component 3 [file mmc3.docx]

| **Importance of components:** | | |  |  |  |  |
| --- | --- | --- | --- | --- | --- | --- |
|  | PC1 | PC2 | PC3 | PC4 | PC5 |  |
| Standard deviation | 1.3273 | 1.2808 | 0.988 | 0.766 | 0.18687 |  |
| Proportion of Variance | 0.3523 | 0.3281 | 0.1952 | 0.1174 | 0.00698 |  |
| Cumulative Proportion | 0.3523 | 0.6804 | 0.8757 | 0.993 | 1 |  |
|  |  |  |  |  |  |  |
| **Rotation (loading)** | |  |  |  |  |  |
|  | PC1 | PC2 | PC3 | PC4 | PC5 |  |
| Pb | 0.234229 | 0.72961 | 0.036672 | -0.16162 | 0.620758 |  |
| Cr | -0.51231 | 0.43584 | 0.25557 | -0.51334 | -0.46771 |  |
| Ni | 0.617881 | 0.369184 | -0.2907 | 0.116558 | -0.61954 |  |
| Cd | -0.47225 | 0.375996 | -0.09803 | 0.789464 | -0.0524 |  |
| As | -0.27908 | -0.00659 | -0.91609 | -0.27115 | 0.096573 |  |
|  |  |  |  |  |  |  |
| **PC scores** |  |  |  |  |  |  |
| sample ID | PC1 | PC2 | PC3 | PC4 | PC5 | Site |
| 19 | 1.980128 | 0.961334 | -1.04961 | -0.00368 | 0.153278 | A |
| 20 | 2.263925 | 0.974937 | -0.20369 | 0.252474 | -0.03023 | A |
| 21 | 2.362681 | 1.103552 | -0.24599 | 0.234601 | -0.18752 | A |
| 40 | 0.348148 | -2.1954 | 0.27931 | 0.677968 | -0.00641 | B |
| 41 | 0.381082 | -2.20724 | 0.269389 | 0.707616 | 0.077091 | B |
| 42 | -0.05941 | -2.21753 | -1.00298 | 0.294879 | 0.135192 | B |
| 61 | -1.12809 | -0.3947 | -1.97571 | -1.35336 | -0.16635 | C |
| 62 | -0.23154 | -0.37671 | 0.748257 | -0.50486 | -0.43013 | C |
| 63 | -0.5267 | -0.43629 | 0.899448 | -0.61684 | -0.16735 | C |
| 76 | -0.14136 | 0.485994 | 0.86976 | -0.80493 | 0.199423 | D |
| 77 | -0.2897 | 0.333419 | 0.922988 | -0.81118 | 0.247681 | D |
| 78 | -0.19364 | 0.396439 | 0.882798 | -0.79169 | 0.203082 | D |
| 85 | -1.45736 | 1.149711 | 0.663228 | 1.163199 | 0.008565 | E |
| 86 | -2.11409 | 1.300411 | -1.67923 | 0.570004 | 0.084948 | E |
| 87 | -1.19407 | 1.122075 | 0.622042 | 0.985795 | -0.12128 | E |
|  |  |  |  |  |  |  |
|  | Pb | Cr | Ni | Cd | As |  |
| Center | 46.27779 | 2.607629 | 2.486999 | 0.384495 | 0.543326 |  |
| Scale | 18.70203 | 1.462365 | 3.110112 | 0.798223 | 1.038833 |  |

**Table S3. Importance of components, Rotation and PC scores for PCA analysis (bee venom samples)**

**Table S4. Importance of components, Rotation and PC scores for PCA analysis (forager bees’ samples)**

| **Importance of components:** | | |  |  |  |  |
| --- | --- | --- | --- | --- | --- | --- |
|  | PC1 | PC2 | PC3 | PC4 | PC5 |  |
| Standard deviation | 1.1115 | 1.0711 | 1.0259 | 0.9078 | 0.8606 |  |
| Proportion of Variance | 0.2471 | 0.2295 | 0.2105 | 0.1648 | 0.1481 |  |
| Cumulative Proportion | 0.2471 | 0.4765 | 0.687 | 0.8519 | 1 |  |
|  |  |  |  |  |  |  |
| **Rotation (loading)** | |  |  |  |  |  |
|  | PC1 | PC2 | PC3 | PC4 | PC5 |  |
| Pb | 0.654062 | -0.16984 | -0.13712 | -0.60023 | -0.40532 |  |
| Cr | -0.45276 | -0.36862 | -0.50708 | -0.51907 | 0.364083 |  |
| Ni | -0.01832 | -0.73586 | -0.23959 | 0.502769 | -0.3847 |  |
| Cd | 0.264842 | -0.5265 | 0.598202 | -0.06324 | 0.53927 |  |
| As | 0.544735 | 0.128773 | -0.55571 | 0.336915 | 0.514152 |  |
|  |  |  |  |  |  |  |
| **PC scores** |  |  |  |  |  |  |
| sample ID | PC1 | PC2 | PC3 | PC4 | PC5 | Site |
| 1 | -2.5717 | -2.49361 | -2.15359 | -1.54281 | 0.735476 | A |
| 2 | -2.31171 | -1.97043 | -1.76774 | -1.47103 | 0.698985 | A |
| 3 | -1.89272 | -2.15731 | -2.66603 | -1.18939 | 1.340237 | A |
| 4 | -0.13348 | -0.70086 | -0.74847 | 0.781001 | 0.002184 | A |
| 5 | -0.99652 | -1.2714 | -0.40773 | 0.306863 | -0.56458 | A |
| 6 | -1.06645 | -0.93065 | -0.3652 | -0.06222 | -0.28802 | A |
| 7 | -0.32463 | 0.521654 | 0.349261 | -0.88873 | -0.12389 | A |
| 8 | 0.215189 | 0.523595 | -0.28182 | 0.39626 | 0.463644 | A |
| 9 | -0.65166 | 0.131677 | 0.319553 | -0.1131 | -0.22011 | A |
| 10 | 0.00702 | 0.495049 | -0.35065 | 0.113817 | 0.581233 | A |
| 11 | -0.63109 | 0.564149 | 0.468916 | -0.39155 | -0.00632 | A |
| 12 | -0.25731 | 0.700367 | -0.12119 | -0.30287 | 0.608103 | A |
| 13 | -0.68915 | 0.35005 | 0.353196 | -0.33691 | -0.05206 | A |
| 14 | 1.885382 | 0.497849 | -2.63076 | 1.485271 | 2.351598 | A |
| 15 | -0.45994 | -0.71488 | -0.71838 | 0.377791 | 0.068169 | A |
| 16 | -0.51191 | 0.324832 | -0.04463 | -0.27339 | 0.285029 | A |
| 17 | -0.89674 | 0.226263 | 0.134438 | -0.60777 | 0.139936 | A |
| 18 | -0.87484 | 0.346521 | 0.190088 | -0.65708 | 0.179078 | A |
| 22 | 1.233033 | 0.542832 | -1.04328 | 1.352655 | 0.939381 | B |
| 23 | -0.34997 | 0.623912 | 0.732374 | 0.053608 | -0.32607 | B |
| 24 | 0.095337 | 0.5435 | 0.212537 | 0.459518 | 0.000973 | B |
| 25 | 0.242223 | 1.057052 | 0.231746 | 0.21386 | 0.380108 | B |
| 26 | -0.01147 | 1.208641 | 0.565245 | -0.09173 | 0.246917 | B |
| 27 | 1.678813 | 1.380108 | -1.23964 | 1.114013 | 1.727733 | B |
| 28 | -0.3468 | 0.75114 | 0.773798 | -0.03332 | -0.25956 | B |
| 29 | -0.34256 | 0.921459 | 0.829252 | -0.14969 | -0.17052 | B |
| 30 | 0.180108 | 0.920134 | 0.251956 | 0.261345 | 0.260088 | B |
| 31 | -0.34421 | 0.855223 | 0.807686 | -0.10443 | -0.20514 | B |
| 32 | 0.503149 | 1.128467 | -0.24647 | 0.264455 | 0.858103 | B |
| 33 | -0.18443 | 0.630411 | 0.551972 | 0.178931 | -0.18621 | B |
| 34 | 1.035787 | 1.175389 | -0.60226 | 0.753348 | 1.094334 | B |
| 35 | -0.40956 | 0.653413 | 0.68934 | -0.07139 | -0.23493 | B |
| 36 | -0.34013 | 1.019352 | 0.861125 | -0.21657 | -0.11934 | B |
| 37 | -0.34128 | 0.972955 | 0.846019 | -0.18487 | -0.14359 | B |
| 38 | -0.342 | 0.944165 | 0.836645 | -0.1652 | -0.15865 | B |
| 39 | -0.41611 | 1.033658 | 0.799174 | -0.35902 | -0.01604 | B |
| 43 | -0.42363 | -2.33536 | -0.23113 | 2.0755 | -1.87317 | C |
| 44 | -0.3984 | -1.32179 | 0.098873 | 1.38299 | -1.34328 | C |
| 45 | 0.587352 | -1.15584 | -0.93043 | 2.039814 | -0.44656 | C |
| 46 | -0.37347 | -0.3201 | 0.425014 | 0.698592 | -0.8196 | C |
| 47 | 2.697094 | -1.01275 | -0.55042 | -1.48784 | -2.17822 | C |
| 48 | 0.254876 | -0.22911 | -0.23631 | 1.127666 | -0.25544 | C |
| 49 | -0.43395 | 0.219173 | 0.536104 | 0.201693 | -0.4449 | C |
| 50 | 1.744134 | -4.59552 | 5.16756 | 0.01527 | 3.581335 | C |
| 51 | 3.372543 | -2.02521 | 0.744994 | -1.42461 | -0.07993 | C |
| 52 | 0.750577 | -1.04995 | -1.07318 | 2.093465 | -0.25869 | C |
| 53 | -0.57687 | -1.40053 | -0.08078 | 1.130018 | -1.16288 | C |
| 54 | -0.02542 | -1.28377 | -0.29933 | 1.64892 | -1.01643 | C |
| 55 | 2.579918 | 0.03525 | -0.87605 | -0.73876 | -0.64843 | C |
| 56 | -0.16797 | -0.21071 | 0.236849 | 0.782953 | -0.5951 | C |
| 57 | -0.39064 | -0.28528 | 0.420608 | 0.643448 | -0.77875 | C |
| 58 | -0.36627 | -0.0309 | 0.519173 | 0.501003 | -0.66841 | C |
| 59 | 0.345458 | -0.52483 | 1.067372 | -0.09844 | -0.31344 | C |
| 60 | 1.108536 | -0.24882 | 0.199995 | -1.14279 | -1.44418 | C |
| 64 | 3.299814 | -0.18176 | -1.19221 | -2.41545 | -1.05977 | D |
| 65 | 0.670613 | -0.2563 | -0.10172 | -1.66407 | -0.96285 | D |
| 66 | 2.496208 | 0.021379 | -1.53556 | -1.24597 | 0.051237 | D |
| 67 | -0.77115 | -0.04164 | -0.88093 | -0.69828 | 0.932407 | D |
| 68 | -1.23954 | 0.300766 | -0.14205 | -1.25768 | 0.611512 | D |
| 69 | -1.28261 | 0.028876 | -0.26224 | -1.13499 | 0.514925 | D |
| 70 | -0.57763 | 0.500212 | 0.416238 | -0.47869 | -0.04678 | D |
| 71 | -0.02498 | 0.544244 | -0.43423 | -0.01159 | 0.722981 | D |
| 72 | -0.67106 | 0.410598 | 0.387377 | -0.34946 | -0.04122 | D |
| 73 | -0.45111 | 0.695866 | 0.665988 | -0.17444 | -0.15926 | D |
| 74 | -0.53714 | 0.598113 | 0.561208 | -0.25295 | -0.10542 | D |
| 75 | -0.16315 | 0.658877 | 0.131477 | -0.02076 | 0.274159 | D |
| 79 | 0.923124 | 0.734879 | -0.9355 | 0.828814 | 1.098094 | E |
| 80 | -0.18022 | 0.414259 | 0.440473 | 0.319399 | -0.25915 | E |
| 81 | -0.37141 | 0.752624 | 0.752778 | -0.07716 | -0.22785 | E |
| 82 | -0.19862 | 0.206031 | 0.417809 | 0.466033 | -0.41108 | E |
| 83 | -0.49971 | 0.516216 | 0.56899 | -0.12839 | -0.19779 | E |
| 84 | -0.083 | 1.068122 | 0.586717 | -0.05289 | 0.125592 | E |
|  |  |  |  |  |  |  |
|  | Pb | Cr | Ni | Cd | As |  |
| Center | 0.095714 | 0.196899 | 1.649033 | 0.003664 | 0.446914 |  |
| Scale | 0.285454 | 0.334037 | 1.414614 | 0.022573 | 0.67016 |  |
